# Infections With Extracellular Trypanosomes Require Control by Efficient Innate Immune Mechanisms and Can Result in the Destruction of the Mammalian Humoral Immune System

**DOI:** 10.3389/fimmu.2020.00382

**Published:** 2020-03-11

**Authors:** Stefan Magez, Joar Esteban Pinto Torres, Emmanuel Obishakin, Magdalena Radwanska

**Affiliations:** ^1^Laboratory for Biomedical Research, Ghent University Global Campus, Incheon, South Korea; ^2^Department of Biochemistry and Microbiology, Ghent University, Ghent, Belgium; ^3^Laboratory of Cellular and Molecular Immunology, Vrije Universiteit Brussel, Brussels, Belgium; ^4^Biotechnology Division, National Veterinary Research Institute, Vom, Nigeria; ^5^Department of Biomedical Molecular Biology, Ghent University, Ghent, Belgium

**Keywords:** trypanosomes, innate immunity, cytokine, antibody, immunosuppression

## Abstract

Salivarian trypanosomes are extracellular parasites that affect humans, livestock, and game animals around the world. Through co-evolution with the mammalian immune system, trypanosomes have developed defense mechanisms that allow them to thrive in blood, lymphoid vessels, and tissue environments such as the brain, the fat tissue, and testes. Trypanosomes have developed ways to circumvent antibody-mediated killing and block the activation of the lytic arm of the complement pathway. Hence, this makes the innate immune control of the infection a crucial part of the host-parasite interaction, determining infection susceptibility, and parasitemia control. Indeed, trypanosomes use a combination of several independent mechanisms to avoid clearance by the humoral immune system. First, perpetuated antigenic variation of the surface coat allows to escape antibody-mediated elimination. Secondly, when antibodies bind to the coat, they are efficiently transported toward the endocytosis pathway, where they are removed from the coat proteins. Finally, trypanosomes engage in the active destruction of the mammalian humoral immune response. This provides them with a rescue solution in case antigenic variation does not confer total immunological invisibility. Both antigenic variation and B cell destruction pose significant hurdles for the development of anti-trypanosome vaccine strategies. However, developing total immune escape capacity and unlimited growth capabilities within a mammalian host is not beneficial for any parasite, as it will result in the accelerated death of the host itself. Hence, trypanosomes have acquired a system of quorum sensing that results in density-dependent population growth arrest in order to prevent overpopulating the host. The same system could possibly sense the infection-associated host tissue damage resulting from inflammatory innate immune responses, in which case the quorum sensing serves to prevent excessive immunopathology and as such also promotes host survival. In order to put these concepts together, this review summarizes current knowledge on the interaction between trypanosomes and the mammalian innate immune system, the mechanisms involved in population growth regulation, antigenic variation and the immuno-destructive effect of trypanosomes on the humoral immune system. Vaccine trials and a discussion on the role of innate immune modulation in these trials are discussed at the end.

## Introduction

Human African Trypanosomosis (HAT) and Animal Trypanosomosis (AT) are parasitic diseases that are caused by unicellular protozoan organisms of the class Kinetoplastida. While classic HAT is a disease that is strictly confined to specific areas of sub-Sahara Africa, AT is a disease that is found on all continents except Antarctica. Most pathogenic livestock trypanosomes are salivarian trypanosome that are being transmitted by the bite of a bloodsucking vector. Often, transmission is the result of insect bites, but mechanical incidents such as vampire bat bites or injury-induced blood contact can also be a source of disease transmission. Human trypanosome infections are in 98% of all cases the consequence of the *Trypanosoma (Trypanozoon) brucei gambiense* parasite that occurs in West- and Central-Africa. The remaining infections are caused by zoonotic *Trypanosoma brucei rhodesiense* parasites. HAT is geographically restricted to sub-Sahara Africa as *T. brucei* parasites strictly depend on completion of their lifecycle inside their final host, i.e., the tsetse (Glossinidae) fly. Occasional HT infections have been reported outside Africa. These cases are classified as non-classic Human Trypanosomosis and are the result of *Trypanosoma (Trypanozoon) evansi* infections. So far, such infections have only been reported in Asia, even though the causative agent is also present in South America, Africa, northern islands of Oceania and even occasionally occurs in Europe. In contrast to HAT, animal trypanosomosis is caused by a wide range of trypanosomes. To start, *T. evansi* is the most widely distributed animal trypanosome. This is a consequence of the fact that the parasite has a very wide transmission vector range combined with a very wide mammalian host range. It has evolved beyond the need to complete its lifecycle inside the tsetse fly and hence has been able to move away from the sub-Saharan tsetse belt. Secondly, *Trypanosoma (Dutonella) vivax* is a livestock parasite that is found in sub-Sahara Africa and South America. Also here, the transmission vector range extends beyond the insect reservoir, as additional mechanical transmission modalities contribute to disease spread. Thirdly, *Trypanosoma (Nannomonas) congolense* is restricted to the African continent. This parasite is mainly transmitted by blood sucking insects from the families of Glossinidae, Tabanidae, Chrysops, Atylotus, and Muscidae. The host tropism for this parasite ranges from domestic animals with economic importance, to wildlife species that harbor parasites as a reservoir, without suffering from severe infection-associated pathology. The latter makes it virtually impossible to envisage the total eradication of this parasite from the African continent. Finally, *Trypanosoma (Trypanozoon) brucei* is considered a vector transmitted parasite with veterinary importance, although the “disease” contribution of this parasite to the overall problem of trypanosomosis is minor. Besides the vector borne trypanosomes, there is also *Trypanosoma (Trypanozoon) equiperdum*, which is a sexually transmitted parasite that causes a deadly disease in equines. Over the years this parasite has occasionally caused havoc in Southern Europe due to illegal or ill-controlled import of infected livestock. The geographic distribution of all HAT and AT causing parasites and general aspects of the immunopathology related to their infections were recently reviewed by Radwanska et al. (1).

In general, host immune responses and immune evasion strategies of salivarian trypanosomes are comparable, although specific host-parasite interactions do govern unique aspect of infection depending upon both parasite and host species. The general hallmarks of infections can be considered to be (i) activation of the innate immune system helping initial parasitemia control, but also driving immune pathology, (ii) antigenic variation of the parasite attempting the evasion of the antibody immune response, and (iii) modulation and destruction of the adaptive immune system (2). In addition, there is the strict regulation of parasite proliferation in the mammalian host that contributes to the intricate control of the host-parasite interaction, as well as the tissue tropism that contributes to the specific characteristics of disease outcome (3). This includes the density-dependent differentiation from the proliferative bloodstream form into the non-proliferating parasite form that is adapted to transmission to the insect vector. In case of HAT, there are two extra components that add to this equation. The first is that during chronic infection, parasites cross the blood brain barrier where they can hide from most of the immune system, causing the deadly neurological pathology outcome of *sleeping sickness*. The second component is the presence of lytic factors in human serum. These offer protection against all trypanosomes except *T. b. gambiense, T. b. rhodesiense* and possibly some isolates of *T. evansi*, which have acquired resistance mechanisms that defeat the lytic human serum activity. While some consider these lytic factors to be part of the humane innate immune system, the biochemical trypanolytic activity of human serum factors should probably more adequately be described as a secondary moonlighting effect of the serum protein APOL1, rather than an immune function *sensu stricto*. All these interactions will be further discussed in this review.

With HAT being a zoonotic infection, control of human infection by itself can result in the temporary reduction of victim numbers, but in order to sustain disease control efforts, the animal reservoir component needs to be considered as well. That means that a sub-Saharan landmass of about 10 million km^2^, encompassing 36 countries, where trypanosomes and their transmission vectors are present, should not be considered trypanosomosis-free until the entire livestock herd as well as the wildlife game reservoir is cleared from infections. This is obviously an impossible task to achieve. Hence, for now, disease control relies on detection and treatment of positively diagnosed HAT and AT cases, mass-treatment of economically important animals, and efforts to control insect vectors. Under these conditions, access to a protective anti-trypanosome vaccine would be a huge advantage, and since the 1970's efforts have been undertaken to try and deliver such intervention strategy. Unfortunately, nearly 50 years later, not a single vaccine candidate has made it through a successful field trial. All published efforts undertaken in this context, as well as possible explanations of pitfalls of the applied protocols will be discussed in this review. In short, most vaccine efforts have been focusing on identifying antigens and protocols with a good antibody-induction potential, being driven by the idea that B cell mediated antibody production should be the ultimate weapon against extracellular trypanosomes. However, this strategy does not take into account two issues. The first is that that antibody induction upon antigen stimulation, and B cell memory recall potential during the initiation of infection, are two different immunological concepts. It is the latter mechanism that fails during trypanosomosis. Secondly, trypanosomes have acquired a range of tools to avoid the dangers posed by antibodies as they have adopted to survive in the confined environment of the host blood. This is not a hostile environment, this is where they “live.” One way to possibly circumvent these issues is to focus on T cell epitope vaccination rather than B cell epitope targeting (4). Indeed, while the latter is “anticipated” by the parasite and has driven the evolutionary development, triggering T cell memory prior to infection might be a way to boost the immunological “help” in a way that so far has not been part of the immunological pressure that drives the trypanosome genetic drift. T cell vaccination would indeed speed up the initiation of B cell help as well as activate innate inflammatory anti-trypanosome responses upon initial parasite encounter. This idea is based on the fact that trypanosome surface proteins harbor several more conserved hidden epitopes that are not truly immunogenic as such. However, once presented in an MHC-II context by B cells to vaccine induced Th cells, these epitopes could function as the kick-start intermediate link that could tip the balance in favor of the host. Aspects of vaccine failure, vaccine interference by trypanosomes, and ideas dealing with T cell vaccination are being addressed later-on in this review.

## Trypanosome Growth And Reduce Parasite Fitness Is Controlled By Innate Immune Responses

Sterile immunity against mammalian trypanosomosis never seems to occur, especially not in murine model for salivarian trypanosomosis. The term “resistance” that is often used to describe for example C57BL/6 mice that show a relatively good early parasite load control, and prolonged control of infection, refers only to parasitemia control itself. It does not cover the fact that these mice suffer from severe immunopathology, neither does the term cover the fact that all trypanosome infected C57BL/6 mice will succumb to infection. Similarly, “susceptible” mice such as BALB/c mice, have increased levels of peak parasitemia, often accompanied by a shorter survival time, but exhibit signs of reduced inflammatory pathology as compared to the more resistant strains. It is here that the innate response against infections functions as a double edge sword. While an early IFNγ-mediated inflammatory response will help to control the onset of parasitemia as well as the height of peak parasitemia, it will also be the source of infection-associated anemia, liver inflammation, and the destruction of the adaptive immune response. Hence, it needs to be counterbalanced by a later IL-10 response in order to avoid the occurrence of sever pathology. As the aspects of trypanosomosis-associated immunopathology have recently been reviewed elsewhere (5, 6) this review will rather focus on the immune drivers able to trigger the innate responses that help to control infection. When it comes to the latter, there is a consensus about the fact that IFNγ is the key factor that determines very early-on in infection, whether or not the host will exhibit a relatively good level of parasitemia control (7). This cytokine is first produced by NK cells (8), after which NKT and T cells take over (9). This crucial role for IFNγ was first shown by infecting C57BL/6 mice as well as IFNγ and IL-4 knock-out mice (on the same genetic background) and demonstrating that in the absence of IFNγ, mice became highly susceptible to *T. b. rhodesiense* infection (7). This susceptible phenotype was directly linked to the inability of macrophages to provide a proper anti-VSG response in the absence of IFNγ activation, and their failure to engage in the optimal pro-inflammatory response crucial for the control of early-stage parasitemia (10). This early response to trypanosomosis was shown to be MyD88 dependent (11) and TLR9 dependent (12). Interestingly, exposure of susceptible BALB/c mice to CpG ODN improved their relative resistance, increased their pro-inflammatory cytokine production upon trypanosome exposure and elevated anti-trypanosome B and T cell responses, corroborating the importance of this pathway. At the molecular level, the IFNγ stimulation of the macrophage compartment offers an improved response to the recognition of the trypanosome VSG-GPI anchor (13). The structure of this anchor has been described in detail for both *T. brucei* (14) and *T. congolense* (15) and was show to have a poly-mannose carbohydrate unit that is at the core of the recognition by macrophages. Recognition of this GPI glycosyl core by IFNγ stimulated macrophages results in the induction of TNF, which is the second cytokine that is key to the optimal control of an early peak parasitemia. This was shown in both *T. brucei* (16) and *T. congolense* (17) infections, using TNF knock-out mice as well as treatment with anti-TNF neutralizing antibodies (18). This TNF effect works in conjunction with nitric oxide (NO), which was also demonstrated to be crucial for trypanosomosis control. The role of NO by itself merits special mentioning here as it is also involved in multiple aspects of trypanosomosis-associated immunopathology. First, NO was shown to be the main immunosuppressive macrophage product that suppresses T cell mediate immunity and hence hampers the buildup of T cell memory capacity early on in infection (19). NO is also able to exert a direct cytostatic activity on *T. brucei* (20) and *T. congolense* (21). For the latter, differences in susceptibility levels between mouse strains was directly linked to their NO response during infection (22). *In vivo* antibody based killing of trypanosomes also required the presence of IFNγ-mediated NO production, most likely affecting parasite fitness during peak parasitemia (23). Interestingly, also TNF has been suggested to negatively affect parasite fitness, showing a direct trypanolytic effect on some trypanosomes. However, for various trypanosome stocks, this effect was not confirmed in the absence of other inflammatory effector molecules such as NO. What does seem to be a more universal observation is the fact that TNF signaling during trypanosomosis is mainly mediated by the TNF receptor-I (TNFp55) (17). As this receptor also drives the induction of immunopathology, this signaling pathway itself can lead to a dual outcome. Interestingly, hosts that respond to acute experimental trypanosomosis by shedding of their soluble TNF receptor-II (TNFp75) suffer much less infection-associated pathology as this mechanism can serve as a two-stage scavenger/release principle. Indeed, during acute TNF production, the presence of the soluble p75 receptor causes the neutralization of the cytokine by forming a receptor-target complex in the circulation. Once the concentration of TNF starts to drop, the complex dissociates and ensures the prolonged presence of the cytokine in the circulation, helping the prolonged control of parasitemia (24).

Most knowledge covering natural and innate resistance to trypanosomosis is derived from experimental mouse infections, which often are initiated by the intra-peritoneal injection of bloodstream form parasites. However, when intradermal infections are initiated with such parasites, *T. congolense* as well as *T. brucei* experiments have shown that the minimum dose required to obtain infection is 100 times higher compared to the dose required for initiation of infection by intraperitoneal injection. This observation suggest that there are improved innate anti-parasite response that govern surveillance at the dermal exposure sites (25). These results could be relevant for vector transmitted infections, as at least in cattle it was shown that it is the innate arm of the immune system, and not the hematopoietic system, that controls parasite growth (26). One obvious cellular player than comes to mind when considering dermal immunology is the presence of neutrophils. Surprisingly, a recent study addressing the role of these innate cells during the onset of infection has shown that they have an infection-enhancing effect, rather than an inhibitory function (27). While the functional role of these cells has not been addressed in other immune sites, their cell number increases dramatically in the spleen of trypanosome infected mice (28).

As for the innate control of human infection, the situation is not totally clear. For a long time, HAT has been called a “lethal” infection, but now the notion is being accepted that there are individuals that carry long-lasting asymptomatic infections. Here, blood parasite levels are so low they are impossible to detect (29). Hence, it could be that a very efficient innate control mechanism manages to subdue infection from the start and does not allow the parasite ever to reach detectable levels in these HAT cases. Till now, it is not clear which mechanisms exactly would drive this resistance as very little HAT-tolerance data is available. While IL-8 has been suggested to be a possible mediator here, TNF and IL-10 have both been associated with disease development (30). An IL-17/Th17 driven susceptibility pathway that has been suggested to occur in *T. congolense* infections (31), has so far not been corroborated to contribute to human HAT related immunopathology or regulation of tolerance.

## Non-Immune “Innate” Primate Serum Factors Can Offer Biochemical Protection Against Trypanosomosis

While the innate immune system of the vertebrate host is a crucial determinant during the onset of a trypanosomes infection, there are additional components that will determine whether or not the initiation of infection is successful. This relates to the fact that the biochemical composition of certain vertebrate host plasma simply does not allow particular parasites to grow. Indeed, while *T. b. gambiense* and *T. b. rhodesiense* are considered severe human pathogens, *T. b. brucei* parasite are unable to survive in human blood due to the particular biochemical composition of human serum. Hence, the latter poses no danger to human health. The fact that the biochemical composition of host serum can determine whether or not a given trypanosome species can successfully initiate an infection, was first proposed when the growth of *T. b. brucei, T. b. gambiense*, and *T. b. rhodesiense* were analyzed in the presence of normal (non-immune) human serum (NHS). The fact that *T. b. brucei* is lysed by NHS through an immune-independent mechanism explained why this parasite is unable to infect humans. This trypanolytic biochemical activity is not just found in human serum, but is also present in the blood of other nonhuman primates such as gorilla, baboon, mandrill and sooty mangabey serum, but not chimpanzee, orangutan and macaque (32). In human serum, two different trypanolytic fractions have so far been identified, i.e., TLF1 and TLF2. While both fractions are part of the high-density lipoprotein (HDL) subfraction, they differ in some aspects of their composition, with TLF2 containing a natural IgM fraction (33). What they do have in common is that they contain two compounds that are now generally been agreed upon as necessary for the lytic activity, i.e., Apolipoproteins L1 (APOL1) as the actual lytic compound, and haptoglobin-related protein (Hpr) needed for receptor recognition. While some have called the TLFs as “novel components of the innate immunity” (34), one might argue that they are actually not true immune actors and that the trypanolytic activity of these HDL fractions are biochemical activities that support protection against certain trypanosome species due to a fortunate moonlighting function. Within the context of this review, it is however interesting to highlight the defense principles that TLFs offer against trypanosomes, and how both *T. b. rhodesiense* and *T. b. gambiense* have acquired individual mutations that in turn offers protection against TLFs. First, the primary discovery of how *T. b. rhodesiense* is able to grow in NHS, while *T. b. brucei* is not, started in the 1980s with a long-term genetic analysis project comparing different *T. b. rhodesiense* stocks with various levels of NHS resistance and *T. b. brucei* stocks. This work culminated in the discovery of SRA (serum resistance antigen), a homolog of the variant surface glycoprotein (VSG) that makes up for the bulk of the trypanosomes surface coat and is crucial for protection against antibody-mediated immune attacks. SRA is constitutively expressed by all *T. b. rhodesiense* parasites when growing in human serum, as “the” molecule conferring resistance to the non-immune lysis by NHS (35). Using *T. b. rhodesiense* SRA as a fishing tool, APOL1 was subsequently isolated from NHS and ultimately proven to be the biochemical compound that carries the actual lytic activity killing activity (36). Today, there is wide consensus over the fundamentals of the APOL1-SRA interaction, the principles that drive APOL1-mediated trypanosome killing in general, and the way *T. b. rhodesiense* neutralizes the killing activity, i.e., by capturing and degrading APOL1 during the process of endocytosis (37). This, in turn, neutralizes the membrane pore forming capacity that APOL1 exerts on intracellular membranes (38). Interestingly, the way that *T. b. gambiense* developed resistance against TLFs is much more complicated and appears to combine multiple mechanisms. First, as *T. b. gambiense* does not have SRA or an SRA homolog, it does not appear to have the capacity to directly neutralize the biochemical lytic activity of the TLFs. In contrast, the resistance mechanism of *T. b. gambiense* involves (i) a reduced binding activity of TLF for its receptor, resulting in a reduced TLF uptake, (ii) increased cysteine protease activity involved in APOL1 breakdown, and (iii) a role for TgsGP, a VSG-like *T. b. gambiense* specific glycoprotein (39). The latter was elegantly proven by generating a *T. b. gambiense* TgsGP knock-out parasite line as well as a rescue mutant, and showing that survival in human serum directly correlated to the presence of the targeted gene (40). Interestingly, while these data show that *T. b. gambiense* went through several evolutionary steps to become an omnipotent infective *T. brucei* parasite, some primates have undergone APOL1 evolution so that it confers resistance against *T. b. gambiense*. This is the case for the West African Guinea baboon that lives in a *T. b. gambiense* endemic area, and has a gene encoding for an APOL1 variant that confers protection against both *T. b. brucei* and *T. b gambiense* (41). These examples are a clear indication of how the arms race between parasites and host innate defense system can shape the genome of both partners, and how even non-immune “innate” responses can be a key factor in disease resistance or susceptibility.

## Trypanosomes Control Their Population Density By Quorum Sensing

The innate host immune responses obviously play a crucial role in the control of the initial onset of trypanosome infections. However, it is known that the peak blood parasite load in experimental infections is not just linked to host immune factor activity, but also depends on intrinsic characteristics of the parasite. Indeed, early experiments using nu/nu T cell knock-out mice, and later experiments in μMT B cell deficient mice have indicated that maximum parasite density during peak parasitemia is not significantly affected by either of these cell populations and appears to be regulated by a quorum sensing mechanism of the parasite itself. In *T. brucei*, this mechanism seems to drive the differentiation from dividing slender form parasites into non-dividing stumpy form parasites, hence halting the proliferation of the population (42). By showing that the system was operational *in vitro*, it was concluded at the time that this sensing mechanism was independent of host factors, and was dependent on the secretion by the parasite of an elusive stumpy inducing factor (SIF) (43). SIF was shown to trigger cell cycle arrest in the G_1_/G_0_ phase preceding cell differentiation, potentially involving a cAMP signaling pathway. Without proper identification of the exact nature of SIF, the story of quorum sensing in trypanosomes remained silent for about 15 year, until the recent use of a genome-wide RNAi library screen identified the signaling components that drive stumpy formation. Candidate genes were subsequently validated, confirming their role in density sensing *in vivo*, leading to the identification of the putative RNA-binding protein RBP7 as a key component in both quorum sensing and cell-cycle arrest (44, 45). Interestingly, the quorum sensing observed in *T. brucei* is also observed in *T. congolense* although in the latter, density-dependent cell-cycle arrest does not result in actual stumpy form formation and does not follow exactly the same gene regulation profile (46). It does however prepare the parasite for transmission to its definite insect host. Under field conditions, *T. brucei* and *T. congolense* coinfections are not uncommon, raising the question how the quorum sensing factors of one parasite would affect the other. The results of combined experiments showed the systems are indeed similar as the *T. congolense* genes can complement the pathway in *T. brucei*. In addition, conditioned culture medium from *T. congolense* promotes stumpy formation of *T. brucei in vitro*, and *T. congolense* co-infection accelerates differentiation to stumpy forms in *T. brucei* (47).

Most recently, the investigation of the quorum sensing mechanism in *T. brucei* has delivered an exciting breakthrough than can explain a number of results that previously had been difficult to align with the view that SIF is purely dependent on the parasite, without any involvement of the host. Indeed, while trypanosomes do not have G protein-coupled receptors the Matthews laboratory identified a GPR89-family protein that regulates stumpy formation in *T. brucei* (48). *Tb*GPR89 is expressed on the surface of proliferating slender form parasites and is able to sense the presence of SIF, which so far was only identified as a small <500 Da non-proteinaceous heat-stable factor. Based on its structure, *Tb*GPR89 was predicted to be an oligopeptide transporter and given that the secretome of *T. brucei* contains a number of oligopeptidases, the most straight-forward explanation for the density-dependent stumpy transformation is the fact that as trypanosomes accumulate in their environment (*in vitro* or *in vivo*), so does the oligopeptidase activity. This would subsequently create a pool of peptide breakdown products from surrounding proteins of which specific oligoptide compounds can be detected by the parasite. As this signal increases, the parasite slows down its own growth, avoiding the killing of its host by hyper parasitemia and at the same time preparing itself for transmission to its insect vector (49). There are still arguments that would defend the existence of a second density independent stumpy development pathway in *T. brucei* (50), in which expression site regulation is the key to the transformation process, but only the GPR89 driven system would solve the question that has been raised by several immunologists when looking at the quorum sensing mechanism of trypanosomes, i.e., how is it possible that when comparing parasitemia of a cloned parasite in different mice with different genetic backgrounds, the peak parasitemia level is defined by the genetic background of the host and not the parasite? Indeed, when comparing the growth of identical pleomorphic *T. b. brucei* AnTat 1.1E parasites in a range of different mouse strains (including knock-out strains), peak parasitemia density directly correlates to the intrinsic inflammatory bias of the host. Mice with a natural low inflammatory induction potential such as BALB/c or C3H/HeN mice show a much higher peak parasitemia density than mice with high inflammatory induction capacity such as CBA/Ca or C57BL/6 mice (24). Also, when B cell deficient mice on a C57BL/6 or BALB/c background are challenged with identical *T. brucei* parasites, the peak parasitemia density is very different depending on the genetic background of the host, but not affected by the presence or absence of antibodies (51), once again showing that the stumpy differentiation and the control of peak parasitemia density is affected in the first place by differences in host genetics. The answer could be that extracellular oligopeptidases from inflammatory immune cells and inflammation damaged tissue contributes to the formation of the peptides that make up the SIF-pool. From an evolutionary point of view that would mean that trypanosomes have “learned” not just to sense their own density, but at the same time can sense the inflammatory state of the host. When either hyper-parasitemia, or hyper-inflammation risks killing the host, the parasite population goes into growth arrest and counts on vector transmission for ultimate survival.

## Trypanosomes Avoid Antibody-Mediated Killing By Dynamic Surface Coat Interactions

Once trypanosomes have established a successful infection, it is obvious that due to the extracellular nature of these infections, antibodies are going to be the main immune component that the parasite will have to deal with. It is generally accepted that the pressure exerted by antibodies has resulted in the fact that extracellular trypanosomes have developed a very sophisticated mechanism of antigenic variation, which allows the switching of the antigenic type of their glycoprotein coat at regular time intervals. The mechanisms that are being used by trypanosomes to do this are remarkably similar to the mechanism that the host uses to generate antibody diversification (52). Indeed, while both trypanosome “cells” and B cells use a system of mono-allelic expression to ensure that only one VSG (trypanosomes) or one B cell receptor (BCR) is expressed by a single cell at any given time, both the entire trypanosome population within the host, as well as the entire hosts' B cell population, both exist out of multiple different clones, representing an overall heterogenous population. In addition, proliferation in combination with (somatic) hyper-mutation allows both VSGs and BCRs to play a game of hide-and-seek, ensuring chronic infections and resulting in the continued undulating parasitemia occurrence that is typical for natural trypanosome infection. The occurrence of antigenic variation in human trypanosome infections and the implications for sero-diagnostics has been covered by others (53), and both the molecular mechanisms (54) as well as the *in vivo* dynamics of antigenic variation of the trypanosome surface coat have been described and reviewed in detail in the past (55), and hence are not the target of this review. Recently, the diversity of VSG genes in *T. brucei* was addressed during chronic infections, using the application of long read sequencing (56). Obtained data have confirmed the existence of a VSG repertoire that cannot be exhausted by immune pressure, due to the continuous generation of new mosaic genes that had been reported earlier (57). In addition, this recent data confirms that every parasitemia peak consists of multiple trypanosome populations expressing multiple VSGs, that sometimes are closely related. Finally, this data also demonstrates that there is significant expressed diversity that follows a semi-reproducible pattern over time, when infections in different mice are compared (56). Hence, in the context of this review it is interesting to recapitulate how the antibody arm of the mammalian immune system interacts with the trypanosome surface, and how the trypanosome manages to evade immune destruction by the antibody response. First, it is important to stress that for most trypanosome species, the VSG coat consist out of a densely packed layer of surface glycoproteins, stacking an estimated 10^7^ identical copies of homodimer molecules evenly distributed over the parasite surface. Hence, this coat has long been considered as an “impenetrable” barrier. More recent structural data have shown however that the real barrier function that would protect the plasma membrane is only being provided by the glycosylation present at the base of the VSG, and that multiple invariant surface proteins are actually fully accessible to the host antibody response. These new data highlight the gap in our understanding of how the VSG coat really works (58). The gap in understanding is further highlighted by the observation that trypanosomes that express two different VSG variants at the same time, an event that could take place at the time point where one VSG coat is being replaced by a new coat, fail to trigger an efficient B cell response (59). Hence, while expressing a mosaic coat could be a good way to evade antibody-mediated elimination, this is not what trypanosomes have evolutionary developed as escape strategy. Interestingly, a VSG mosaic coat does not deliver intrinsic constraints that would hinder BCR crosslinking and subsequent B cell activation. Indeed, it has been estimated that the VSG density of intact *T. brucei* parasites is 20 times higher than the density required for BCR cross-linking. So, even if a VSG mosaic coat consists out of two antigenically district variants, each of the variants should in theory be able to mediate regular B cell activation, which is apparently not what happens (60). Yet, experimental infections in distinct mouse strains, as well as their F1 offspring, have shown that susceptibility to infection in terms of limited parasitemia control and shortened survival can be linked to the impaired capacity to mount a proper anti-VSG response. Interestingly, F1 generations showed that the capacity to mount a good anti-VSG antibody response is inherited as a dominant trait, while survival time remained similar to the susceptible parental strain (61). These data showed that antibody-mediated parasitemia control and disease susceptibility in terms of survival are controlled by different mechanisms. Furthermore, these finding were corroborated by studying *T. b. rhodesiense* infections in mouse chimera models, indicating a role for IgM in parasitemia peak control but not survival (62). With the arrival of mouse knock-out technology, the role of antibodies in trypanosomosis control was reassessed in various models. First, it was shown that in experimental *T. b. brucei* infections, IgMs do play a role in peak parasitemia clearance (but not the control of the peak height itself) in the relatively virulent AnTat 1.1E pleomorphic needle transmission model. In contrast no obvious role was observed in the control of the low virulent TSW196 field isolate, or during tsetse fly initiated AnTat infections (51). Interestingly, B cell deficient μMT mice (either on a susceptible BALB/c background or a more resistance C57BL/6 background) failed to clear peak parasitemia levels. Hence, one can conclude that while IgMs do help to control more virulent infections, there are other antibodies that can take over the role in parasite clearance when IgMs are absent. In a model for *T. b. rhodesiense*, it was shown that IgM antibodies have a better trypanosome agglutination potential, but that the IgG fraction provided a better protective response in serum transfer experiments (63). This could be related to the better tissue penetrating capacity of the latter, which could be important during natural and chronic HAT infections. Next, the role of IgMs was addressed in experimental *T. congolense* infections using the chronic Tc13 model. Here it was found that while C57BL/6 μMT mice were completely susceptible and succumbed to infection following an uncontrolled first peak of parasitemia, IgM deficient mice survived on average nearly 4 months, similarly to the fully immune competent controls. Interestingly, as already outlined in the section above on innate anti-trypanosome responses, infection control in this model was shown to be dependent on the presence of an INFγ/NO/TNF inflammatory environment. Most likely, this environment reduces parasite fitness, allowing antibody-mediated clearance without the strict requirement for the participation of IgM antibodies (23). It could be argued however that as the *T. b. brucei* setting, intrinsic parasite virulence is also a factor here and that in case of a more virulent *T. congolense* infection, there could be a supporting role for IgMs in parasite clearance. This has not been tested so far. For experimental *T. evansi* infections the results appear to be the somewhat opposite of what was reported for *T. congolense*. Here, both C57BL/6 μMT mice and IgM deficient mice showed a very high level of susceptibility, being unable to control the first peak of parasitemia. Hence, in these infections IgGs are not able to take over the protective role of IgM (64). Whether or not these results were affected by the intrinsic virulence level of the stabilates used, remains to be tested. Finally, in case of *T. vivax*, infections were done in μMT mice as well as a number of cytokine deficient mice, but not in IgM deficient mice. Results available so far show that the combination of anti-trypanosome antibodies and an inflammatory immune environment, in particular the presence of TNF, is needed for proper parasitemia control, very similarly to the results described for *T. congolense* (65).

So far, IgMs appear to play a crucial role in all trypanosome models at least in the control of more acute infections. Hence, it is interesting to speculate on the exact functional mechanisms involved here, which could involve a role for the Fcα/μ receptor. This Fc receptor has been shown to be important for the endocytosis of IgM opsonized microbes (66), although it has not been studied in the case of trypanosomosis. With no evidence for a role of complement-mediated lysis of trypanosomes in *in vivo* infection control (this is discussed in detail in the section below), it appears indeed that phagocytosis, in particular by Kupffer cells, of opsonized parasites is the only way that antibodies can help in the clearance of peak parasitemia numbers (67). Additional data suggest that the presence of an inflammatory environment aids this process, most likely due to the fitness reducing effects of TNF and NO, either by themselves or when combined. Interestingly, by artificially blocking VSG synthesis using an RNAi approach, it was shown that the process of macrophage phagocytosis was much more efficient (68). This finding suggests that VSG recycling is a key process in the defense of the parasite against the attack by the mammalian antibody response. This hypothesis is supported by an observation dating back to 1979, when D. J. Barry first described that upon exposure of trypanosomes to homologous antiserum, VSGs moves to the flagellar pocket in a temperature dependent process (69). The presence of a phospholipase C enzyme that could release soluble VSG by cleaving its GPI anchor in the flagellar pocket, was later suggested also to be part of the VSG-membrane recycling system (70). The detailed recycling pathway of VSG was subsequently described by two independent groups (71, 72), showing that trypanosome have an extremely fast rate of endocytosis resulting in the turnover of their VSG coat within 12 min and the formation of 6–7 clathrin-coated vesicles per second, that bud from the flagellar pocket and deliver the membrane content to RAB5-positive early endosomes. Next, VSG is recycled through RAB11-positive recycling endosomes that return the VSG to the surface. It is now believed that trypanosomes use this system not only for the uptake of nutrients contained in the fluid phase of the vesicles and bound to exposed membrane receptors, but that in addition it allows the parasite to “clean” the coat from bound antibodies. This protects the parasites from damaging antibody functionality as long as the cells are in an optimal metabolic state. This hypothesis was very elegantly shown by Engstler et al. when they reported that Ig-VSG membrane immune complexes are passively sorted to the posterior cell pole, where they are endocytosed through the flagellar pocket. This process requires forward cell motility and results from the hydrodynamic forces that are encountered due to the directional “swimming” motion of the trypanosomes (73). It was shown that *in vitro* this molecular flow movement protected trypanosomes from complement-mediated lysis. The same principle would also protect the trypanosome from efficient antibody-mediated phagocytosis *in vivo* as surface-bound antibodies would function as molecular sails that would direct immune compromised VSGs toward the flagellar pocket for “cleaning” or replacement (74). It is obvious that as the immune system would mount an increasing antibody response with time, this system would become saturated and eventually fail. However, this delay in antibody-mediated destruction possibly offers an extended time window during which antigenic variant can take place. This will increase the chance that a surface VSG appears that is distinct enough to avoid recognition of the parasite by existing circulating antibodies, allowing the survival of some parasites within the population.

## Trypanosomes Avoid Antibody-Mediated Killing By Destruction Of The Host B Cell Compartment

Despite the importance of antibodies in the control of extracellular trypanosome parasites, there is relatively little data available describing the actual role and immunobiology of B cells in either experimental mouse trypanosomosis or natural human/cattle infections. In cattle, B cell responses have been compared between trypanotolerant N'Dama and susceptible Boran cattle, in order to try and link humoral immune response to parasitemia control. While it has already been mentioned that in cattle actual parasitemia control was found to be in large regulated by the innate immune response (26), these comparative studies did show that trypanotolerant cattle were able to produce higher anti-VSG IgG serum responses (75). In addition, these animals had more circulating lymphocytes that could be activated *in vitro* to undergo differentiation into IgM- and IgG secreting cells. In contrast, trypano- susceptible cattle showed higher frequencies of spleen IgM secreting cells producing antibodies that were not directed toward the trypanosome VSG coat. Interestingly, in both *T. congolense* and *T. vivax* cattle infection, CD5^+^ B1 cells were found to be the main source of poly-reactive IgM responses, producing antibodies that bind to β-galactosidase, ovalbumin and ferritin (76). This polyclonal B cell activation is considered to be part of the “immunosuppression” that characterizes trypanosome infections, as it *de facto* reduces the chance for an efficient specific immune response to be developed against crucial parasite antigens. Polyclonal B cell activation has also been reported in experimental infections with *T. b. brucei* and *T. b. rhodesiense* in mice (77). While the latter report linked this immunological phenomenon to the presence of the VSG, the exact mechanism driving the activation has so far not been unraveled. Besides polyclonal activation, there are two other B cell biology observations that merit attention in the context this review, i.e., the non-classic regulation of Ig class switching, and the destruction of the B cells compartment that constitutes the main pathology problem for the immune system during chronic infections. Unusual Ig class switching was first described in a study that measured anti-VSG isotype profiles during *T. b. rhodesiense* infections in mice. Here is was found that both fully immune competent C57BL/6 mice as well as T cell deficient nu/nu mice were able to mount a very similar switched anti-VSG response that mainly consisted out of IgG1, IgG2a, and IgG3 antibodies. This occurred in the presence of IFNγ and IL-2, but the absence of IL-4 and IL-5. However, when repeating these measurements in IL-4 knock-out mice, a significant decrease in IgG1 titers was observed, indicating a role for this cytokine in the regulation of B cell activity, independent of antigen specific Th1 or Th2 cells (78). In the same context, IgG switching was also observed in experimental *T brucei* infections in both WT and nu/nu BALB/c mice, with IgG1, IgG2a, IgG2b, and IgG3 isotype antibodies being produced independent of conventional T cell help (79). Interestingly, in both models the lack of T cell help did not seem to hamper parasitemia control, while the lack of Il-4 and the subsequent capacity to drive an IgG1 B cell differentiation pathway also did not affect parasitemia control. One example where B cell regulation was shown to be a crucial arm of parasitemia control was in chronic *T. congolense* infections in mice. Here Bam32, responsible for downstream signaling linked to BCR crosslinking, was shown to be crucial for prolonged parasitemia control, albeit redundant for early peak parasitemia elimination (80). This observation aligns with the finding that *T. congolense* infections cause a sustained disruption of the B cell homeostasis in bone marrow and spleen, and that the virulence index of different stocks in experimental mouse models correlates with the potential to drive B cell destruction (81). When it comes to immune destruction of the B cell compartment, this has also been documented in detail for *T. vivax* (82) and in more detail for *T. brucei* (83). In the latter model it has been shown that the early loss of B cells is linked to an excessive inflammatory IFNγ response (84), can involve the NK cell mediated killing activity (85), is observed both at the level of the bone marrow and spleen (86) and takes several weeks to be restored after drug treatment of experimentally infected mice (87). Also here, infection-induced B cell destruction seems to be associated to parasite virulence as mice that are infected with very chronic *T. b. gambiense* parasites do not seem to suffer the same B cell depletion problem as those infected with a much more acute *T. b. brucei* strain (88).

## Serum Complement Factors Mediate Parasitemia Control *In vivo*, But Not Trypanolysis

Immunoglobulins only have a limited range of function they can perform by themselves. In essence, good antibodies are proteins that bind their target with high affinity and specificity. For trypanosomosis control, both antibody-mediated phagocytosis (discussed above) and antibody-mediated complement lysis are quoted throughout the literature. However, four decades of complement research in the context of trypanosomosis have shown that “lysis” is not the mechanism that mediates parasitemia control during *in vivo* infection. The reason for the persistence of this misconception about complement lysis of trypanosomes has probably to be attributed to the fact that *in vitro* antibody-mediated trypanolysis assays are being used as diagnostic tools for the detection of active infections. Indeed, when cultured or purified trypanosomes are incubated *in vitro* with high amounts of complement-rich guinea pig serum and plasma from infected individuals or animals, trypanosomes tend to lyse over time proportionate to the amount of anti-trypanosome antibodies present in the donor serum (89). This phenomenon has been very clearly described in the 1970's in the correct context of the particular *in vitro* conditions for *T. congolense* and *T. brucei*, and the authors also immediately discussed the limitations of the observation with respect to the *in vivo* situation (90). Also the involvement of the alternative activation of the complete cascade under *in vitro* conditions with the role of C3 and the formation of covalently immune complexes between released soluble sVSG and C3b has been described, without any claim that this would represent the system that controls parasitemia *in vivo* (91). In fact, over the years, a wealth of data has been published that shows that parasitemia control *in vivo* occurs totally independent from the lytic complement cascade, and that trypanosomes have a number of tools at their disposition to efficiently prevent complement-mediated lysis of actively dividing bloodstream form parasites. Maybe the most compelling data in this context comes from experimental infections in animals that simply do not have the capacity to activate the full complement cascade due to genetic mutations. For example, AKR mice as well as B10.D2/oSnJ mice are both deficient for C5. Hence, while the complement cascade can be initiated in these mice with the formation of the opsonin C3b molecule, the cascade cannot progress toward the formation of the membrane pore forming 10–16 C9 complex. Therefore, the fact that B10.D2/oSnJ mice are able to control successive waves of *T. b. rhodesiense* infection proves that complement-mediated lysis is not crucial in the process of either peak parasitemia control, or peak parasitemia clearance (92). Similarly, these C5-deficient mice were capable of controlling *T. musculi* infections (93), as well as *T. congolense* infections (94). Also AKR mice were used to study the progression of infection in models for both *T. b. rhodesiense* (95) and *T. vivax* (65), showing again that peak parasitemia control as well as peak parasitemia clearance occurs in the absence of C5, and by consequence in the absence of complement-mediated trypanolysis. The lack of C5 involvement in trypanosomosis control is further corroborated by the fact that *T. congolense* infections hardly affected plasma C5 levels in a range of different mouse models, including highly susceptible and highly tolerant strains (96). Interestingly, the latter study did report that trypanosome infections resulted in a rapid decline of C3 in all strains tested. This confirmed earlier results that had shown that while C8 levels are unaffected during infection C1, C1q, and C3 levels all decrease in plasma of *T. congolense* infected cattle (97) as well as in *T. vivax* infected cattle (98). Finally, it is important that these experimental mouse and cattle data also reflect the situation in humans. Indeed, while *T. b. gambiense* does activate C3 and binds C3b on its surface when incubated with human serum, it does not result in the formation of C5 or the polymerization of C9. Hence, trypanosomes are not lysed as the complement cascade does not continue beyond the establishment of C3 convertase (99).

So, could there still be a role for complement in the control of trypanosomosis? The “yes” answer relates to the fact that besides lysis, the initial stages of complement are crucial for the generation of the C3b component, a powerful opsonin that binds covalently to its target (100). By interaction with plasma Factor H and I, C3b can be converted into iC3bi (or C3bi) which is a target for the CR3 (CD11b/CD18) receptor involved in pathogen phagocytosis (101). In case of *T. congolense* infections it has been described that mice with effective prolonged parasitemia control and low parasitemia peaks (C57BL/6) have a faster and greater C3b production than mice that intrinsically fail to control the infection with the first 2 weeks of a parasite challenge (BALB/c) (102). This activity correlates with the finding that the CR3 receptor is a major player in the endocytosis of *T. congolense* parasites after opsonization of the parasite by IgM antibodies (103). Interestingly in an innate immune context is the finding that CR3-mediated phagocytosis is also directly linked to macrophage TNF production, an inflammatory cytokine that as described above has a profound negative impact on trypanosome fitness. Complement associated phagocytosis has also been described for *T. brucei* (104), and more recently the use of C3 knock-out mice confirmed a role for complement in peak parasitemia clearance during *T. brucei* infections (85). All these data confirm older results showing (i) that early stage complement cascade activation is important for parasite clearance (105), (ii) that hepatic phagocytosis of trypanosomes depends on the presence of C3, and (iii) that *in vivo*, no intravascular lysis of parasites is observed during events of immune clearance (106). However, simply to illustrate the complexity of the immune system, it should be said that in the presence of IgM opsonization of *T. brucei* parasites, treatment of mice with CVF (cobra venom factor) that neutralizes C3, did not ablate the capacity of macrophages to phagocytose trypanosomes. This shows that in immune competent mammals, multiple mechanism are in place to deal with peak parasitemia clearance (107).

Taken that C3b/iC3b deposition on the VSG surface coat can result in hepatic phagocytosis, the trypanosome must have defense mechanisms in place in order to ensure that this system does not lead to population elimination. As already outlined above, rapid VSG circulation comes into play here, as a defense system against any host compound binding to the surface (108) and as a tool for complement surface clearance in particular (109). Secondly, the densely packed nature of the VSG coat itself offers physical protection against any possible complement-mediated lysis (110). Finally, it has been proposed that trypanosomes have two more defense systems in place that could help the fight against host complement. First, trypanosomes were shown to express a homolog of the *Leishmania* GP63 surface protease that has been shown to confer resistance to complement-mediated lysis (111). Secondly, *T. brucei* was shown to bind human complement regulatory protein C4BP. Acquisition of this factor on the surface of pathogens is correlated to the downregulation of complement activation (112). Hence, together these mechanisms provide the trypanosome with adequate tools to confer resistance to complement mediate lysis, but at the same time allow the immune system to clear parasites trough phagocytosis in the liver. The latter would be particularly helpful when parasite fitness is reduced due to ongoing inflammatory responses triggered by the innate immune system, and during events of parasite growth inhibition that result from the quorum sensing mechanisms described above.

## T Cells Contribute To The Maintenance Of An Anti-Trypanosome Innate Inflammatory Environment

Since the very beginning of trypanosome immunology research, T cells have been considered crucial for proper trypanosomosis control. How and why this is the case, strongly depends on the model studied and the definition of infection control. For example, when calves were depleted from CD4^+^ T cells and trypanosome control was studied, the effects observed were strongly dependent on the intrinsic trypanotolerance of the breed. While T cell depletion always had a very severe negative effect on antibody production, this did not impact parasitemia control in trypanotolerant N'Dama cattle. In contrast, the same treatment had a profound negative impact on parasitemia control in susceptible Boran cattle (26). This finding suggests that while T cell mediated responses can help to bring parasitemia under control in certain conditions, there are T cell independent factors that can be responsible for intrinsic trypanotolerance. CD8^+^ T cell depletion in susceptible Boran cattle did not affect parasitemia control (113). N'Dama/Boran chimera experiments in cows confirmed that intrinsic parasitemia control was controlled by a hematopoietic independent mechanism, while disease control, i.e., anemia development, was in contrast dependent upon the hematopoietic system.

In mice, control of trypanosome infectious does not exactly mirror the observations of cattle, but there are parallels. A first interesting observation is the T cell deficient nu/nu mice on either BALB/c or C57BL/6 background have no problems in controlling parasitemia in comparison to fully immune competent mice (51, 79). In fact, when it comes to pathology control, C57BL/6 mice that lack T cell altogether, or just CD8^+^ T cells, suffer less from infection-associated anemia (9), and have a prolonged lifespan in case of nu/nu mice when infected with *T. b brucei* or *T. b. rhodesiense* (9, 114). This dichotomy between parasitemia control and pathology/survival most likely reflects the complex role T cells play in (i) sustaining the innate inflammatory environment needed for proper parasitemia control, (ii) the immunopathology associated with prolonged inflammation, and (iii) in case of CD4^+^ T cells, their role in helping a T cell dependent anti-trypanosome antibody response. As also discussed above, antibodies involved in trypanosomosis control in mice comprise both a T cell independent and T cell dependent fraction. Indeed, while even nu/nu mice are able to raise VSG specific antibody responses, fully immune competent mice often—but not always—show a better response (115). Interestingly, in fully immune competent mice T cell responses to trypanosome VSG are not limited to the hypervariable regions but also cover the less variable regions of the N-terminal domain that are inaccessible for antibodies. At the same time however, it appears that during experimental infections no T cell are raised against epitopes located in the relatively invariant C-terminal part of the VSG (116). This opens an interesting hypothesis for future vaccine attempts which is also discussed further in this paper: could it be that trypanosomes evolved to have an immune evasion mechanism that prevents exposing conserved epitopes in an MHCII context, so that during infection no T cell memory is developed against such structurally important domains? While these domains are constrained with respect to sequence variation due to their functional importance for VSG structure, they are buried at the base of the VSG and hence are relatively safe from antibody recognition. If, however such epitopes were to successfully recognize by the T cell immune compartment after antigen processing, they could serve according to the hapten-carrier concept as CD4^+^ T helper cell generators that would provide help to all anti-VSG B cells, against all surface exposed epitopes. This would negatively affect the fitness of the trypanosome. Hence, while such negative selective pressure would favor the acquisition of non-immunogenic conserved regions, the artificial induction of T cell responses against these epitopes prior to infection might tip the balance in favor of the host, in a way that trypanosomes have not been evolutionary prepared for (4).

Beside the possible help to B cells, it is clear that T cells provide crucial help to the maintenance of a correct cytokine environment, needed to help control parasitemia through innate related mechanisms. Indeed, it has been shown that in C57BL/6 mice that exhibit a relatively good parasitemia control, infection control is associated with the induction of Th1 CD4^+^ responses, characterized by the production of IFNγ and IL-2 and the absence of IL-4 (117). The importance of this response for macrophage activation and parasitemia control was confirmed by the fact that C57Bl/6 IFNγ knock-out mice are highly susceptible for trypanosome infections, as already indicated in the section that covered the innate protection against the parasite. In contrast, IL-4 knock-out mice showed a phenotype that was identical to fully immune competent mice (7). This drive toward Th1 differentiation has been correlated directly to the splenic DC antigen presentation of VSG that coincides with IL-12 secretion (118). At first, these results might seem conflicting to the results observed in trypanosome infected nu/nu mice, where the latter are characterized by a normal parasitemia control. It is however important to emphasize the fact that infection-induced IFNγ production does not solely result from the presence of activated CD4+ T helper cells. In particular during early infection, it was indeed shown that the first cells to respond with a significant IFNγ secretion are NK cells, subsequently followed by NKT cells and only later by Th1 CD4^+^ T cells and CD8^+^ T cells. Hence, it is possible that as far as the early need for IFNγ is concerned, nu/nu mice have a compensatory mechanism in which the NK/NKT compartment delivers the correct cytokine environment to control early parasitemia by a combination of inflammatory cytokines and T cell independent antibodies (9). Finally, one line of thinking that also could explain in part the results observed in overall comparative mouse infection, is that so called “wild type” mice undergo an immune suppression of T cells that makes them *de facto* closely resemble functional T cell deficient mice. In this case, trypanosome infected WT mice would indeed functionally resemble nu/nu mice in some respects, despite the presence of T cells in the lymphoid circulation.

## Experimental Anti-Trypanosome Vaccines Have So Far Not Been Translated Into Applicable Vaccines

In 2011, we published a comprehensive overview of anti-trypanosome vaccine data that was available at the time (119). We concluded that while there is a fair amount of “promising” results in the literature, there is little prospect of seeing a successful field applicable vaccine any time soon. Ten years later, a number of additional results have been published, but it appears that the latter statement still stands. An overview of the currently reported anti-trypanosome vaccine data is provided in Table 1.

**Table 1:** Summary of vaccine candidates reported in literature.

**Type of vaccine**	**Antigen**	**Antigen preparation**	**Boosts/host**	**Time gap boost-challenge**	**Parasite load**	**Outcome**	**References**
Intra-muscular	*T. b. rhodesiense* FP	Parasite isolated	3/cattle	14 days or more	Natural exposure	Partial protection	(120)
I.p.	*T. b. brucei* AnTaR FP	Parasite isolated	3/mouse (Balb/c)	3 weeks	500–10^3^	Partial/no protection	(121)
I.p.	*T. b. brucei* MITat ISG65, ISG75	Recombinant protein	3/mouse (C57bl/6)	11 days	10^4^	No protection	(122)
I.p.	*T. b. brucei* ISG75	Plasmid DNA	1/mouse (Balb/c)	175 days	500	Partial protection	(123)
I.p.	*T. b. brucei* GuTat10.0 Ca^2+^ ATPase TBCA2	Recombinant protein	3/mouse (Balb/c)	6 weeks	10^6^	No protection	(124)
Sub-cutaneous	*T. brucei* (UTRO010291B) Tubulin rich fraction	Parasite isolated	3/mouse (no strain indication)	Not indicated	10^3^-10^5^	Partial (cross-species) protection	(125)
Sub-cutaneous	*T. evansi* (STIB806) b-Tubulin	Recombinant protein	3/mouse (Balb/c)	6 days	10^3^	Partial (cross-species) protection	(126)
Intra-muscular	*T. evansi* (EU483116) β-Tubulin	Plasmid DNA	2/mouse (Swiss albino)	35 days	10^3^	No protection	(127)
Sub-cutaneous	*T. evansi* (EU483116) β-Tubulin	Recombinant protein	2/mouse (Swiss albino)	14 days	10^3^	No protection	(128)
Sub-cutaneous	*T. evansi* (STIB806) Actin	Recombinant protein	3/mouse (Balb/c)	6 days	10^3^	Partial (cross-species) protection	(129)
Sub-cutaneous	*T. congolense* CP1 & CP2	Recombinant protein	4/cattle (Boran)	1 month	Tsetse bite	Improved recovery	(130)
Intra-muscular	*T. b. brucei* Sialidase	Plasmid DNA	1/mouse (Balb/c)	175 days	500	Partial protection	(131)
I.p.	*T. congolense* Sialidase	Recombinant protein	4/mouse (Balb/c)	10–14 days	10^4^	Partial protection	(132)
I.p.	*T. b. brucei* AnTat GPI	GPI-Liposomes	2/mice (C57bl/6 and KOs)	3 weeks	5 × 10^3^	Cross-species anti-pathology	(133)

*I.p., intraperitoneal; FP, Flagellar Pocket; ISG, Invariant Surface Glycoprotein; GPI, glycosylphosphatidylinositol; CP, Cystein proease*.

The rationale behind most anti-trypanosome vaccine efforts is to vaccinate a host with invariant trypanosome proteins or a mix of such targets. Given the role of the flagellar pocket in nutrient uptake, and the presence of a number of surface receptors and invariant surface molecules such as the LDL receptor and the HpHb receptor, Invariant Surface Glycoproteins ISG65 and ISG75, and SRA (134), the FP was targeted in two independent approaches. In one report, cattle were inoculated with crude antigen extracts derived from the FP of *T. b. rhodesiense*, and subsequently exposed through a possible natural challenge with *T. congolense* or *T. vivax* by releasing the animals in their natural field habitat (120). This resulted in a significant drop in infection prevalence at the end of the observation period that lasted 15 months. In a more controlled mouse vaccine study, FP vaccination was conducted using highly purified material from *T. b. brucei* parasites. Borderline protection was observed when mice were challenged with 500 live bloodstream form parasites, with 60% of mice not developing parasitemia for a period of up to 100 days (121). No protection was observed however when mice were challenged with 1,000 parasites or more. Important is that the ultimate idea behind a vaccine is to deliver memory recall responses for prolonged periods of time. In the setting used here, this was not tested as the waiting period between the last boost and the parasite challenge was only 21 days. Hence, immune modulation events, including innate responses triggered by repeated adjuvant exposure, could have contributed to the limited protection observed here in a low-dose challenge setting.

Upon discovery of the invariant trypanosome surface proteins ISG65 and ISG75, they were considered as viable vaccine candidates. While ISG75 was shown to be a good antibody inducer, no protection was observed in a vaccine setting using a homologous parasite challenge, despite the very short 11-day waiting period between the last boost and the actual parasite injection (122). Next, a DNA vaccine approach targeting ISG75 was tested. Here, a single vaccination was followed 175 days later by exposure to a low-dose challenge with 500 *T. b brucei* GVR35 parasites. The results obtained were virtually identical to the mouse FP vaccine low-dose challenge experiment and showed 60% protection against parasitemia for a period of at least 60 days (123). As in the case of the FP vaccination, this outcome was associated with a strong IgG2a antibody presence, indicative of an IFNγ driven cytokine environment. These results do not conclusively point to the involvement of an antibody effector mechanism and could equally well-indicate that biasing the host toward a strong inflammatory mediated immune regulatory environment, favors protection against low-dose parasite exposure, due to a more efficient help to the innate anti-trypanosome immune response. A very similar conclusion can be made for an anti-trypanosome vaccination that used the Ca^2+^ ATPase TBCA2 antigen as a target in BALB/c mice. This trypanosome membrane pump was formulated in a *Vibrio cholera* ghost-based vaccine, after it was identified as one of the conserved FP components (124). This vaccine gave rise to the induction of a prominent IgG2a response accompanied by a strong IFNγ cytokine production. However, in this case the combination of these favorable factors did not prevent the successful onset of parasitemia by *T. brucei* trypanosomes, as the infection was initiated with an extremely high number of 10^6^ parasites.

By far the most often targeted trypanosome antigen is the tubulin protein, and as a variation on the idea, the actin protein (125–129, 135). Both proteins are part of the intracellular cytoskeleton structure and have functions in motility and intracellular organelle transport but are not accessible by the host antibody response. This makes the repeated reports of the successful induction of protection using various protocols very interesting. What all the protocols have in common, is that the waiting period between the last boost and the actual challenge was too short to asses vaccine induced memory, in some cases being limited to 6 days only. Hence, these protocols again assessed the direct effect of immune modulation by the procedure. What all reports also have in common is that none of them attempted to explain how the intracellular trypanosome proteins would have been targeted by the host antibody response in order to provide sterile protection. Once again, the most obvious explanation is that the applied protocol provided an improved inflammatory environment that favored the innate immune control of infection during the onset of parasitemia.

So far, most anti-trypanosome vaccine studied have been performed in mice, for obviously logistical reasons. Several authors have however questioned this approach and have argued that vaccine studies for AT should be conducted in the relevant host. The only molecular target that has been tested in this context is the *T. congolense* cysteine protease congopain, after it was shown that trypanotolerant N'Dama cattle, but not trypanosusceptible Boran cattle, mount and IgG1 response against this target (136). Successfully vaccinated cattle did show increased anti-CP serum antibody titers, as well as anti-VSG titers, coinciding with improved in parasitemia control (130). Next, the immunogenicity of CP was improved by coupling the catalytic domain of the enzyme to α_2_-macroglobulin (137), and various adjuvants were compared leading to the adjuvant choice of Quil A™ for future experiments (138). Important to stress here is that this approach is not an anti-parasite approach, but in the end, it is a way to modulate the host immune response and alleviate the inflammatory-associated immune pathology of trypanosomosis. The molecular mechanism by which this was achieved still remains open for discussion.

Using trypanosome enzymes as vaccine targets has also been applied in a *T. brucei* setting, targeting trans-sialidase (TS). Using TS encoding plasmid DNA, a single-dose vaccine experiment was followed 175 days later by a *T. b. brucei* GVR35 challenge, using 500 parasites. The obtained results show a 40% protection against infection for a period of at least 60 days (132). As the report did not detail a functional antibody analysis, it is difficult to provide a final mechanistic hypothesis for the partial protection. However, given the type of vaccine used (DNA vaccination) one could once again suggest that an IFNγ-driven helper response could be part of the equation, helping both the innate response immediately upon parasite challenge, as well as the IgG2a response that would occur upon B cell reactivation. In a follow-up vaccination approach against *T. congolense*, this time using recombinant TcoTS, protection levels reached 15–40% (131). Here, vaccine-induced memory responses were once again not addressed as the waiting period between the last boost and parasite challenged were limited to <14 days. Hence, this makes it very plausible that the limited level of protection observed was related in part to immune modulation and improved innate responses, rather than just anti-trypanosome antibody induction.

By considering the strategy of anti-pathology vaccination, rather than anti-trypanosome vaccination, our group has taken a radically different approach in the past to find new ways of dealing with the problems of trypanosomosis. After having identified the VSG-GPI anchor as the main driver of inflammatory pathology and macrophage-derived TNF production (13), we approached anti-disease vaccination for *T. b. brucei, T. evansi*, and *T. congolense* by using a liposome-based GPI vaccine. By challenging mice several times with GPI liposomes prior to trypanosome exposure, the infection-associated pathology was reduced and the lifespan of infected mice was significantly increased (133). Interestingly, using B-cell deficient mice, we were able to show that the observed effect was totally independent of antibody function, even though the protocol did result in the successful induction of antibodies in WT animals. These experiments conclusively showed that vaccine induced immune modulation of inflammation was at the core of the protection. The observation that the CD1d molecule was crucial for mediation of the observed protection, indicates a major role for the modulation of the innate immune system (133).

## Trypanosome Infections Ablate The Efficacy Of Non-Related Protective Vaccines

To date, it seems there no data is available that suggests that vaccine induced immunological memory can be recalled upon during ongoing trypanosome infections, in order to help the host to recover from infection. In addition, combined data shows that infection-associated inflammation is detrimental for the host immune system as whole, and as such could affect immune responses against non-related co-infections, or non-related vaccines. In recent years, this question has been addressed using both experimental mouse models and human case studies. In the past however, a significant amount of data has already been published indicating that trypanosomes do compromise the host immune system of natural hosts in general. In addition, there is data available showing that HAT and AT affect antibody titers against non-related disease, which is particularly important when considering antibody-based diagnostic test evaluation in trypanosomosis endemic areas. All these observations have been summarized in Table 2.

**Table 2:** Summary of the detrimental effect of trypanosomosis in non-related vaccines, B cells, and B cell malignancies.

**Parasite**	**Host**	**Disease/vaccine**	**Readout**	**Outcome**	**Functional readout**	**References**
*T. brucei*	Mouse	*Bordetella pertussis*	CFU counts	Increased lung CFUs	Loss of vaccine protection	(82)
*T. b. gambiense*	Human	HIV	Ab titers Diagnostic test	Decrease in specificity	NA	(139)
*T. b. gambiense*	Human	Measles	Ab titers Diagnostic test	Decreased titers	NA	(140)
*T. congolense*/*T. vivax*	Cattle	CBPP	Ab titers/experimental infection	Decreased titers	50% susceptibility	(141)
*T. congolense*/*T. vivax*	Cattle/mice	Louping-ill virus	Ab titers	90% titer reduction	NA	(142)
*T. congolense*	Cattle	Foot-and-mouth virus	Ab titer	Decreased titers	Virus challenge/no effect on protection	(143)
*T. congolense*/*T. vivax*	Cattle	*Brucella abortus*	Ab titers	80–90% titer reduction (*T. congolense*)	NA	(144)
*T. evansi*	Buffalo	*Pastuerella multocida*	Ab titers/inflammation	Decreased titers	NA	(145)
*T. evansi*	Buffalo	*Pasteurella multocida*	Ab titers/lymphocyte proliferation	Decreased titers immuno-suppression	NA	(146)
*T. evansi*	Pig	Classic Swine Fever (CSF)	Ab titer/fever	Decreased titers	Decreased fever	(147)
*T. brucei*	Mouse	*Trichinella spiralis*	Ab titers/eosinophil counts lymphocyte proliferation	Decreased titers immuno-suppression	Decreased worm expulsion	(148)
*T. brucei*	Mouse	*T. brucei*/*homo- heterologous*	Parasite count	Short-lived specific protection	Loss of short-lived protection	(82)

*NA, None applicable*.

The first time that the detrimental effect of experimental mouse trypanosomosis was investigated in detail in a heterologous vaccine setting, was by analyzing the effect of *T. b. brucei* infections on the efficacy of the commercially available DTPa vaccine (Boostrix®), which provides protection against diphtheria, tetanus, and whopping caught infections (83). After having observed a complete and permanent ablation of vaccine-mediated protection by the trypanosome, it was proposed that it should be assessed whether such immune destructive effect could threaten vaccine efficacy in humans. While experimental pathogen re-challenge experiments in humans can obviously not be performed in a HAT setting, two follow-up observations are worth mentioning in this context. First, it was shown that *T. b. gambiense* infections decrease the specificity of antibody detection tests for HIV diagnosis, warning that classic algorithms that are being used for test interpretation on non-HAT individuals, might not be able to provide adequate diagnostic answers in HAT patients, even after treatment (139). Next, it was shown that HAT patients who had been vaccinated against measles had significantly lower antibody serum titers compared to non-HAT individuals. Antibody titers remained low after curative anti-HAT treatment, although they were above the guideline cut-off in healthy individuals (140). However, as was the case for the HIV diagnostics, the measured antibody levels could very well be an over estimation of the actual functional titer. This is also what has been observed in the case of contagious bovine pleuropneumonia (CBPP) vaccinations in cattle. Here it was shown that in *T. congolense* infected animals, a vaccine against CBPP lost 50% of its efficacy, while only a minor drop in antibody titers was observed (141). Such drop in non-trypanosome related vaccine efficacy, due to the presence of animal trypanosomosis, has been observed in models for louping-ill virus protection (142), foot-and-mouth vaccination (143), a cattle vaccine model for *Brucella abortus* (144), immunization against *Pasteurella multocida* (145, 146), classic swine fever (CSF) vaccination (147), and in a vaccine setting for *Trichinella spiralis* (148). Finally, staying within the boundaries of the trypanosome mouse model, we assessed functional anti-VSG memory responses. Here we used a live infection model rather than a vaccine model in which mice were first challenged with low-virulent parasite clone, followed by a secondary infection with a high virulent clone expressing either an identical VSG surface coat is the parasite used in the primary infection (homologous challenge) or a different VSG coat (heterologous challenge). The obtained result showed that while the first infection did provide VSG-specific protection against a homologs challenge, the protection was short-lived and disappeared within 10 days after clearance of the first wave of the primary infection. Using B cell knock-out mice, we showed the temporary protective response relied on T-cell independent antibodies (82). However, by using a battery of cytokine deficient mice including TNF and IFNγ knock-out mice it was obvious that the short-lived protective antibody response only works in an inflammatory environment that is driven by the early innate immune response. This can explain the abolishment of the protection by day 17 post infection, despite the presence of high anti-VSG antibody titers at the time of challenge.

## Conclusions

**Figure 1:**
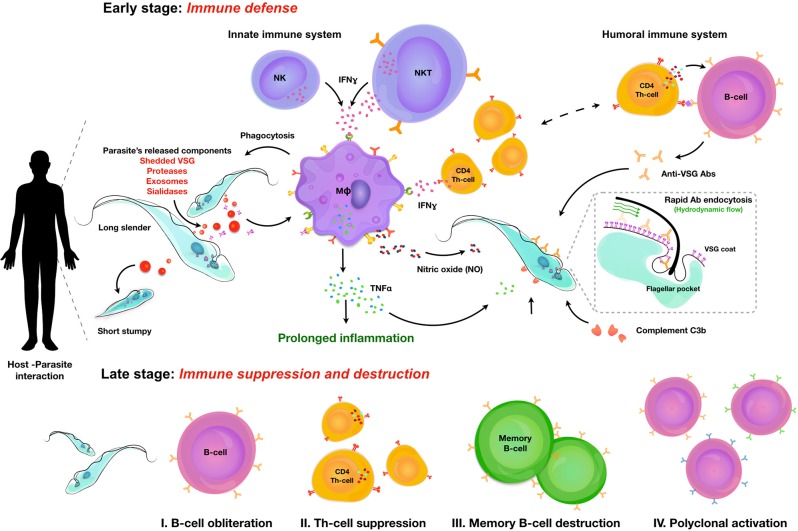
Overview of the crucial aspects of trypanosome-host interactions. Upon infection, trypanosomes are confronted with an early stage inflammatory immune response in which IFNγ is being produced by NK, NKT and subsequently CD4^+^ T cells. This drives the activation of macrophages which in term respond to parasite secreted and released factors, including shedded VSG (Variant Surface Glycoprotein). Activated macrophages produce TNF and Nitric Oxide (NO) that negatively impact on parasite fitness. Antibodies, derived first through T cell independent B cell activation and later through T cell dependent activation, will attack the surface of the trypanosome. Deposition of the C3b complement will also occur. Together, these immune molecules will help in controlling parasitemia, but trypanosomes themselves have defense mechanisms that will help survival. Through lateral VSG movement to the flagellar pocket (FP) antibodies and complement factors are cleared, limiting damage. In order to prevent early killing of the host through parasite overpopulation, trypanosomes have developed quorum sensing, which results in density dependent growth arrest and preparation for vector transmission. Combined, this innate control of infection results in prolonged inflammation with a detrimental outcome for the adaptive immune response. Late stage infections are characterized by B cell destruction, T cell mediated immune suppression, loss of B cell memory recall capacity and irrelevant polyclonal B cell activation.

Extracellular trypanosomes are very successful parasites that today are still expanding their territory. While the terminology “African trypanosomes” is often used synonymously for salivarian trypanosomes, it should be remembered that *T. evansi* was the first extracellular trypanosomes to be discovered, and that this discovery was made in India. Hence, the presence of these parasites should be considered as a global issue, with a possibility of affecting all commercially important livestock mammals as well as game animals. Due to their extracellular nature, and their continuous confrontation by the mammalian immune system, trypanosomes had to acquire multiple defense mechanisms to overcome mainly the dangers posed by host antibodies (Figure 1). Coating their extracellular membrane with a dense layer of VSG glycoproteins offers protection against antibodies, but most likely not in ways that have been suggested in the early days of trypanosome research. First, when during infection the host manages to mount an anti-VSG response, binding immunoglobulins function as molecular sails, and complexed membrane VSG molecules are rapidly transported to the flagellar pocket where they are endocytosed, cleaned and recycled or replaced. Having a *de facto* unlimited library of VSG genes, pseudogenes and the capacity of generating mosaic genes, allows the parasite to switch the antigenic type of the coat and hence avoid complete elimination of the population. Combining this mechanism with the destruction of the host B cell compartment allows the trypanosome to outrun the dangers posed by the host humoral immune response. In addition, multiple studies using mouse knock-out technology, natural mutant mouse strains and AT as well as HAT observations have shown that complement-mediated lysis cannot be used as part of the host defense system, as the complement cascade is blocked from forming C5–C9 components needed for actual lysis. This leaves the host with the system of iC3b-mediated phagocytosis, mainly performed by inflammation-activated Kuppfer cells. In order to protect itself from phagocytosis, the parasite once again uses VSG recycling to prevent total population elimination by clearing iC3b surface deposition. Important however in the case of a parasite, is the fact that killing the host is not a good survival strategy. Hence, trypanosomes have developed a system of quorum sensing that allows them to regulate proliferation in response to parasite density, and most likely is also capable of sensing excessive infection-associated inflammatory tissue damage. While this is crucial to ensure prolonged host survival, it does not prevent the infection of leading to the virtually complete destruction of the host B cell compartment. The latter is most likely the reason for the fact that so far not a single promising laboratory vaccine has been translated into a useful field application. In addition, it explains why many commercial vaccines against unrelated diseases appear to lack efficacy in trypanosome endemic areas. The latter is the case for trypanosusceptible animals, and this could be the foundation for the distinction between susceptibility and tolerance for infection. While trypanotolerance has been described in detail for particular cattle breeds, it is only recently that this phenomenon has now been suggested to also occur in some humans. It would be interesting to see in the future how the B cell compartment of such individuals behaves, as compared to the bulk of the human population that is considered trypanosusceptible. Subsequently, it could be assessed in a HAT context how the relative importance of the innate immune system and the adaptive immune system compare to each other, both in the control of onset of infection as well as in the control of the chronic infection stage.

## Author Contributions

SM and MR: manuscript writing. JP: manuscript reading and figure preparation. EO: manuscript reviewing and reference list editing.

### Conflict of Interest

The authors declare that the research was conducted in the absence of any commercial or financial relationships that could be construed as a potential conflict of interest.
